# The 2018 World Cancer Research Fund (WCRF)/American Institute for Cancer Research (AICR) score and diabetes risk in the Diabetes Prevention Program Outcomes Study (DPPOS)

**DOI:** 10.1186/s40795-022-00596-7

**Published:** 2022-09-21

**Authors:** Marissa M. Shams-White, Ashley H. Tjaden, Sharon L. Edelstein, Sarah Bassiouni, Lisa L. Kahle, Catherine Kim, Xavier Pi-Sunyer, Karla A. Temple, Elizabeth M. Venditti, Jill Reedy, Brandy M. Heckman-Stoddard, DPP Research Group, George A. Bray, Kishore M. Gadde, Iris W. Culbert, Jennifer Arceneaux, Annie Chatellier, Amber Dragg, Catherine M. Champagne, Crystal Duncan, Barbara Eberhardt, Frank Greenway, Fonda G. Guillory, April A. Herbert, Michael L. Jeffirs, Betty M. Kennedy, Erma Levy, Monica Lockett, Jennifer C. Lovejoy, Laura H. Morris, Lee E. Melancon, Donna H. Ryan, Deborah A. Sanford, Kenneth G. Smith, Lisa L. Smith, Julia A. St. Amant, Richard T. Tulley, Paula C. Vicknair, Donald Williamson, Jeffery J. Zachwieja, Kenneth S. Polonsky, Janet Tobian, David A. Ehrmann, Margaret J. Matulik, Bart Clark, Kirsten Czech, Catherine DeSandre, Ruthanne Hilbrich, Wylie McNabb, Ann R. Semenske, Jose F. Caro, Kevin Furlong, Barry J. Goldstein, Pamela G. Watson, Kellie A. Smith, Jewel Mendoza, Wendi Wildman, Renee Liberoni, John Spandorfer, Constance Pepe, Richard P. Donahue, Ronald B. Goldberg, Ronald Prineas, Jeanette Calles, Juliet Ojito, Patricia Rowe, Paul Cassanova-Romero, Sumaya Castillo-Florez, Hermes J. Florez, Anna Giannella, Lascelles Kirby, Carmen Larreal, Olga Lara, Valerie McLymont, Jadell Mendez, Arlette Perry, Patrice Saab, Beth Veciana, Steven M. Haffner, Helen P. Hazuda, Maria G. Montez, Kathy Hattaway, Carlos Lorenzo, Arlene Martinez, Tatiana Walker, Dana Dabelea, Richard F. Hamman, Patricia V. Nash, Sheila C. Steinke, Lisa Testaverde, Denise R. Anderson, Larry B. Ballonoff, Alexis Bouffard, Brian Bucca, B. Ned Calonge, Lynne Delve, Martha Farago, James O. Hill, Shelley R. Hoyer, Tonya Jenkins, Bonnie T. Jortberg, Dione Lenz, Marsha Miller, Leigh Perreault, David W. Price, Judith G. Regensteiner, Helen Seagle, Carissa M. Smith, Brent VanDorsten, Edward S. Horton, Kathleen E. Lawton, Catherine S. Poirier, Kati Swift, Ronald A. Arky, Marybeth Bryant, Jacqueline P. Burke, Enrique Caballero, Karen M. Callaphan, Barbara Fargnoli, Therese Franklin, Om P. Ganda, Ashley Guidi, Mathew Guido, Sharon D. Jackson, Alan M. Jacobsen, Lori Lambert, Sarah Ledbury, Margaret Kocal, Lyn M. Kula, Maureen A. Malloy, Maryanne Nicosia, Cathryn F. Oldmixon, Jocelyn Pan, Marizel Quitingon, Stacy Rubtchinsky, Jessica Sansoucy, Dana Schweizer, Ellen W. Seely, Donald Simonson, Fannie Smith, Caren G. Solomon, Jeanne Spellman, James Warram, Steven E. Kahn, Brenda K. Montgomery, Wilfred Fujimoto, Robert H. Knopp, Edward W. Lipkin, Michelle Marr, Ivy Morgan-Taggart, Anne Murillo, Dace Trence, Lonnese Taylor, April Thomas, Elaine C. Tsai, Samuel Dagogo-Jack, Abbas E. Kitabchi, Mary E. Murphy, Laura Taylor, Jennifer Dolgoff, William B. Applegate, Michael Bryer-Ash, Debra Clark, Sandra L. Frieson, Uzoma Ibebuogu, Raed Imseis, Helen Lambeth, Lynne C. Lichtermann, Hooman Oktaei, Harriet Ricks, Lily M. K. Rutledge, Amy R. Sherman, Clara M. Smith, Judith E. Soberman, Beverly Williams-Cleaves, Boyd E. Metzger, Mark E. Molitch, Mariana K. Johnson, Daphne T. Adelman, Catherine Behrends, Michelle Cook, Marian Fitzgibbon, Mimi M. Giles, Deloris Heard, Cheryl K. H. Johnson, Diane Larsen, Anne Lowe, Megan Lyman, David McPherson, Samsam C. Penn, Thomas Pitts, Renee Reinhart, Susan Roston, Pamela A. Schinleber, David M. Nathan, Charles McKitrick, Heather Turgeon, Mary Larkin, Kathy Abbott, Ellen Anderson, Laurie Bissett, Kristy Bondi, Enrico Cagliero, Jose C. Florez, Kali D’Anna, Linda Delahanty, Valerie Goldman, Peter Lou, Alexandra Poulos, Elyse Raymond, Christine Stevens, Beverly Tseng, Jerrold M. Olefsky, Elizabeth Barrett-Connor, Mary Lou Carrion-Petersen, Madeline Beltran, Lauren N. Claravall, Jonalle M. Dowden, Steven V. Edelman, Robert R. Henry, Javiva Horne, Marycie Lamkin, Simona Szerdi Janesch, Diana Leos, Sunder Mudaliar, William Polonsky, Jean Smith, Jennifer Torio-Hurley, Karen Vejvoda, F. Xavier Pi-Sunyer, Jane E. Lee, David B. Allison, Nnenna Agharanya, Nancy J. Aronoff, Maria Baldo, Jill P. Crandall, Sandra T. Foo, Susan Hagamen, Jose A. Luchsinger, Carmen Pal, Kathy Parkes, Mary Beth Pena, Ellen S. Rooney, Gretchen E. H. Van Wye, Kristine A. Viscovich, David G. Marrero, Kieren J. Mather, Melvin J. Prince, Susie M. Kelly, Marcia A. Jackson, Gina McAtee, Paula Putenney, Ronald T. Ackermann, Carolyn M. Cantrell, Yolanda F. Dotson, Edwin S. Fineberg, Megan Fultz, John C. Guare, Angela Hadden, James M. Ignaut, Marion S. Kirkman, Erin O’Kelly Phillips, Beverly D. Porter, Paris J. Roach, Nancy D. Rowland, Madelyn L. Wheeler, Vanita Aroda, Robert E. Ratner, Gretchen Youssef, Sue Shapiro, Catherine Bavido-Arrage, Geraldine Boggs, Marjorie Bronsord, Ernestine Brown, Wayman W. Cheatham, Susan Cola, Cindy Evans, Peggy Gibbs, Tracy Kellum, Renee Wiggins, Milvia Lagarda, Lilia Leon, Claresa Levatan, Milajurine Lindsay, Asha K. Nair, Maureen Passaro, Angela Silverman, Gabriel Uwaifo, Debra Wells-Thayer, Mohammed F. Saad, Karol Watson, Maria Budget, Sujata Jinagouda, Medhat Botrous, Khan Akbar, Claudia Conzues, Perpetua Magpuri, Kathy Ngo, Amer Rassam, Debra Waters, Kathy Xapthalamous, Julio V. Santiago, Samuel Dagogo-Jack, Neil H. White, Angela L. Brown, Samia Das, Prajakta Khare-Ranade, Tamara Stich, Ana Santiago, Edwin Fisher, Emma Hurt, Tracy Jones, Michelle Kerr, Lucy Ryder, Cormarie Wernimont, Sherita Hill Golden, Christopher D. Saudek, Vanessa Bradley, Emily Sullivan, Tracy Whittington, Caroline Abbas, Adrienne Allen, Frederick L. Brancati, Sharon Cappelli, Jeanne M. Clark, Jeanne B. Charleston, Janice Freel, Katherine Horak, Alicia Greene, Dawn Jiggetts, Deloris Johnson, Hope Joseph, Kimberly Loman, Henry Mosley, John Reusing, Richard R. Rubin, Alafia Samuels, Thomas Shields, Shawne Stephens, Kerry J. Stewart, Lee Lana Thomas, Evonne Utsey, Paula Williamson, David S. Schade, Karwyn S. Adams, Janene L. Canady, Carolyn Johannes, Claire Hemphill, Penny Hyde, Leslie F. Atler, Patrick J. Boyle, Mark R. Burge, Lisa Chai, Kathleen Colleran, Ysela Gonzales, Doris A. Hernandez-McGinnis, Patricia Katz, Carolyn King, Amer Rassam, Sofya Rubinchik, Willette Senter, Debra Waters, Jill Crandall, Harry Shamoon, Janet O. Brown, Gilda Trandafirescu, Elsie Adorno, Liane Cox, Helena Duffy, Samuel Engel, Allison Friedler, Angela Goldstein, Crystal J. Howard-Century, Jennifer Lukin, Stacey Kloiber, Nadege Longchamp, Helen Martinez, Dorothy Pompi, Jonathan Scheindlin, Elissa Violino, Elizabeth A. Walker, Judith Wylie-Rosett, Elise Zimmerman, Joel Zonszein, Trevor Orchard, Rena R. Wing, Susan Jeffries, Gaye Koenning, M. Kaye Kramer, Marie Smith, Susan Barr, Catherine Benchoff, Miriam Boraz, Lisa Clifford, Rebecca Culyba, Marlene Frazier, Ryan Gilligan, Stephanie Guimond, Susan Harrier, Louann Harris, Andrea Kriska, Qurashia Manjoo, Monica Mullen, Alicia Noel, Amy Otto, Jessica Pettigrew, Bonny Rockette-Wagner, Debra Rubinstein, Linda Semler, Cheryl F. Smith, Elizabeth Venditti, Valarie Weinzierl, Katherine V. Williams, Tara Wilson, Richard F. Arakaki, Renee W. Latimer, Narleen K. Baker-Ladao, Mae K. Isonaga, Ralph Beddow, Nina E. Bermudez, Lorna Dias, Jillian Inouye, Marjorie K. Mau, John S. Melish, Kathy Mikami, Pharis Mohideen, Sharon K. Odom, Raynette U. Perry, Robin E. Yamamoto, William C. Knowler, Norman Cooeyate, Mary A. Hoskin, Carol A. Percy, Alvera Enote, Camille Natewa, Kelly J. Acton, Vickie L. Andre, Rosalyn Barber, Shandiin Begay, Peter H. Bennett, Mary Beth Benson, Evelyn C. Bird, Brenda A. Broussard, Brian C. Bucca, Marcella Chavez, Sherron Cook, Jeff Curtis, Tara Dacawyma, Matthew S. Doughty, Roberta Duncan, Charlotte Dodge, Cyndy Edgerton, Jacqueline M. Ghahate, Justin Glass, Martia Glass, Dorothy Gohdes, Wendy Grant, Robert L. Hanson, Ellie Horse, Louise E. Ingraham, Merry Jackson, Priscilla Jay, Roylen S. Kaskalla, David Kessler, Kathleen M. Kobus, Jonathan Krakoff, Jason Kurland, Catherine Manus, Cherie McCabe, Sara Michaels, Tina Morgan, Yolanda Nashboo, Julie A. Nelson, Steven Poirier, Evette Polczynski, Christopher Piromalli, Mike Reidy, Jeanine Roumain, Debra Rowse, Robert J. Roy, Sandra Sangster, Janet Sewenemewa, Miranda Smart, Darryl Tonemah, Rachel Williams, Charlton Wilson, Michelle Yazzie, Raymond Bain, Sarah Fowler, Marinella Temprosa, Michael D. Larsen, Tina Brenneman, Sharon L. Edelstein, Solome Abebe, Julie Bamdad, Melanie Barkalow, Joel Bethepu, Tsedenia Bezabeh, Nicole Butler, Jackie Callaghan, Caitlin E. Carter, Costas Christophi, Gregory M. Dwyer, Mary Foulkes, Yuping Gao, Robert Gooding, Adrienne Gottlieb, Kristina L. Grimes, Nisha Grover-Fairchild, Lori Haffner, Heather Hoffman, Kathleen Jablonski, Steve Jones, Tara L. Jones, Richard Katz, Preethy Kolinjivadi, John M. Lachin, Yong Ma, Pamela Mucik, Robert Orlosky, Qing Pan, Susan Reamer, James Rochon, Alla Sapozhnikova, Hanna Sherif, Charlotte Stimpson, Ashley Hogan Tjaden, Fredricka Walker-Murray, Elizabeth M. Venditti, Andrea M. Kriska, Linda Semler, Valerie Weinzierl, Santica Marcovina, Jessica Harting, F. Alan Aldrich, John Albers, Greg Strylewicz, R. Eastman, Judith Fradkin, Sanford Garfield, Christine Lee, Edward Gregg, Ping Zhang, Dan O’Leary, Gregory Evans, Matthew Budoff, Chris Dailing, Elizabeth Stamm, Ann Schwartz, Caroline Navy, Lisa Palermo, Pentti Rautaharju, Ronald J. Prineas, Teresa Alexander, Charles Campbell, Sharon Hall, Yabing Li, Margaret Mills, Nancy Pemberton, Farida Rautaharju, Zhuming Zhang, Elsayed Z. Soliman, Julie Hu, Susan Hensley, Lisa Keasler, Tonya Taylor, Ronald Danis, Matthew Davis, Larry Hubbard, Ryan Endres, Deborah Elsas, Samantha Johnson, Vonnie Gama, Anne Goulding, Jose A. Luchsinger, Jennifer Manly, Elizabeth Mayer-Davis, Robert R. Moran, Ted Ganiats, Kristin David, Andrew J. Sarkin, Erik Groessl, Naomi Katzir, William H. Herman, Michael Brändle, Morton B. Brown, Jose C. Florez, David Altshuler, Liana K. Billings, Ling Chen, Maegan Harden, Robert L. Hanson, William C. Knowler, Toni I. Pollin, Alan R. Shuldiner, Kathleen Jablonski, Paul W. Franks

**Affiliations:** 1 National Cancer Institute, Bethesda, MD USA; 2 c/o The DPP Coordinating Center, The Biostatistics Center, Milken Institute School of Public Health, The George Washington University, 6110 Executive Blvd., Suite 750, Rockville, MD 20852 USA; 3 Biostatistics Center, Milken Institute of Public Health, George Washington University, Rockville, MD USA; 4 University of California San Diego, San Diego, CA USA; 5 Infomation Management Services, Inc., Rockville, MD USA; 6 University of Michigan, Ann Arbor, MI USA; 7 Columbia University College of Physicians and Surgeons, New York, NY USA; 8 University of Chicago, Chicago, IL USA; 9 University of Pittsburgh School of Medicine, Pittsburgh, PA USA

**Keywords:** Alcohol, Diet, Disease prevention, Obesity, Physical activity, Weight

## Abstract

**Background:**

The 2018 World Cancer Research Fund/American Institute for Cancer Research (WCRF/AICR) 3rd expert report highlights up-to-date Cancer Prevention Recommendations that may reduce burdens of many chronic diseases, including diabetes. This study examined if following a lifestyle that aligns with the recommendations – assessed via the 2018 WCRF/AICR Score – was associated with lower risk of type 2 diabetes in high-risk adults participating in the Diabetes Prevention Program Outcomes Study (DPPOS).

**Methods:**

The Diabetes Prevention Program (DPP) randomized adults at high risk for diabetes to receive a lifestyle intervention (ILS), metformin (MET) or a placebo (PLB) (mean: 3.2 years), with additional follow-up in DPPOS for 11 years (mean: 15 years total). 2018 WCRF/AICR Scores included seven components: body weight, physical activity, plant-based foods, fast foods, red and processed meat, sugar-sweetened beverages, and alcohol; the optional breastfeeding component was excluded. Scores ranged 0-7 points (with greater scores indicating greater alignment with the recommendations) and were estimated at years 0, 1, 5, 6, 9, and 15 (*N*=3,147). Fasting glucose and HbA1c were measured every six months and oral glucose tolerance tests were performed annually. Adjusted Cox proportional hazard ratios (HRs) and 95% confidence intervals (CIs) were used to examine the association of both Score changes from years 0-1 and time-dependent Score changes on diabetes risk through DPP and year 15.

**Results:**

Scores improved within all groups over 15 years (*p*<0.001); ILS Scores improved more than MET or PLB Scores after 1 year (*p*<0.001). For every 1-unit improvement from years 0-1, there was a 31% and 15% lower diabetes risk in ILS (95% CI: 0.56-0.84) and PLB (95% CI: 0.72-0.97) through DPP, and no significant association in MET. Associations were greatest among American Indian participants, followed by non-Hispanic White and Hispanic participants. Score changes from years 0-1 and time-dependent Score changes in ILS and PLB remained associated with lower risk through year 15.

**Conclusions:**

Score improvements were associated with long-term, lower diabetes risk among high-risk adults randomized to ILS and PLB, but not MET. Future research should explore impact of the Score on cancer risk.

**Trial registration:**

Diabetes Prevention Program: NCT00004992; Diabetes Prevention Program Outcomes Study: NCT00038727

**Supplementary Information:**

The online version contains supplementary material available at 10.1186/s40795-022-00596-7.

## Background

The global burden of diabetes is growing. According to the International Diabetes Federation, diabetes affects approximately 451 million people worldwide and may affect 693 million people by 2045 [1]. The cancer burden is similarly growing, with 14.1 million new cases in 2012 and a projected 24 million cases in 2035 [2]. These two non-communicable diseases (NCDs) share mechanistic pathways [3–5] and, given persons with diabetes are estimated to have a 20-25% higher cancer incidence than persons without diabetes [2], a greater emphasis on diabetes prevention could potentially reduce incidence of cancer as well as diabetes.

In 2018, the World Cancer Research Fund (WCRF) and American Institute of Cancer Research (AICR) published updated, evidence-based recommendations focused on lifestyle factors that can reduce cancer risk, as well as other NCDs [6]. The WCRF/AICR Score was created in 2019 to operationalize eight of ten recommendations: 1) maintain a healthy body weight, 2) engage in regular physical activity (PA), 3) eat a diet rich in vegetables, fruits, whole grains, and beans, 4) limit consumption of fast foods and other processed foods high in fat, starches, or sugars, 5) limit consumption of red and processed meats, 6) limit consumption of sugar-sweetened beverages (SSBs), 7) limit consumption of alcohol, and 8) for mothers, breastfeed exclusively if possible for six months [6, 7]. Although cancer risk is an important endpoint, the WCRF/AICR’s lifestyle recommendations also influence other NCDs like diabetes; studies are needed that examine how the Score predicts risk across health outcomes. No studies to date have examined how alignment with the 2018 WCRF/AICR Recommendations affect diabetes risk and diabetes-related outcomes, though evidence supports each of these lifestyle factors’ impact on diabetes risk.

Overweight/obesity, poor diet, and physical inactivity may increase risk for both type 2 diabetes and cancer through similar pathways [6, 8]. Excess intra-abdominal adipose tissue may increase insulin resistance, initiate hyperinsulinemia and chronic low-grade inflammation, and lead to increased pro-inflammatory factors and oxidative stress [2, 9]. PA can also influence body weight and decrease risk of obesity-related cancers, and may improve insulin sensitivity, immunity, and reduce oxidative stress and inflammation [10]. Aside from caloric imbalance, certain dietary factors captured in the WCRF/AICR Recommendations are also strongly associated with obesity and diabetes [11–13]. Fruits and vegetables provide rich sources of antioxidants and, along with dietary fiber intake, may improve insulin sensitivity and secretion and prevent weight gain [13, 14]. Conversely, processed foods have fewer phytochemicals, vitamins, and minerals, which may reduce insulin sensitivity and increase systemic inflammatory markers; processed meats contain higher levels of nitrates, pro-oxidative agents like iron, and advanced glycation end-products, all of which may increase diabetes risk; and sugar-sweetened beverages (SSBs) may impact blood glucose levels, promote hepatic lipogenesis and insulin resistance, and adversely affect regulation of hunger and satiety [11, 13].

The Diabetes Prevention Program (DPP) and follow-up DPP Outcomes Study (DPPOS) investigated if an intensive lifestyle intervention or treatment with metformin in individuals at high-risk could prevent or delay the development of type 2 diabetes [15, 16]. The DPP lifestyle intervention was based on recommendations from the 1995 Dietary Guidelines for Americans and addressed similar areas as the 2018 WCRF/AICR Recommendations [17]. Many past studies investigated how changes in weight, diet quality, and PA individually impact diabetes risk; few studies have examined these lifestyle factors together [18, 19]. DPP provides a unique opportunity to examine how lifestyle changes can affect diabetes risk. This study aimed to examine if 1) following a lifestyle that aligns with the 2018 WCRF/AICR Cancer Prevention Recommendations lowers risk for diabetes in adults at high-risk of type 2 diabetes, 2a) if change in the 2018 WCRF/AICR Score over time affects long-term diabetes risk; and 2b) if any associations differ between those randomized to a lifestyle intervention compared to metformin or placebo.

## Research design and methods

### Study population

The study protocol for DPP is publicly available at https://dppos.bsc.gwu.edu/web/dppos/dpp [20] and the design and methods for both DPP and DPPOS are detailed elsewhere (NCT00004992, NCT00038727) [16, 21–23]. Briefly, DPP was a multicenter, randomized controlled clinical trial that recruited 3,234 participants (68% women, 45% from various ethnic/racial minority groups) from 27 clinical centers across the U.S. (1996-1999). Eligible participants were ≥25 years, had a body mass index (BMI) ≥24 kg/m^2^ (≥22 kg/m^2^ for Asian/Pacific Islanders), and had plasma glucose concentration between 5.3-6.9 mmol/L (95-125 mg/dL) in the fasting state and 7.8-11.0 mmol/L (140-199 mg/dL) two hours following a 75g oral glucose tolerance test (OGTT). Participants were excluded from this secondary analysis if they were missing dietary data (*n*=74), waist circumference (WC) or PA (*n*=4), or had energy outliers (*n*=9) at baseline. Outliers were defined as values more than two interquartile ranges above the 75th or below the 25th percentile on the logarithmic scale. The rate of missing data was low (~70% had dietary and visit data at 15 years) and did not differ among treatment groups; missing data were assumed to be missing at random. The final analytic cohort included 3,147 participants (see Supplemental Figure 1). Participants without year 1 dietary data available were excluded from analyses assessing Score change from baseline to year 1 (*N*=247).

### DPP and DPPOS study designs

Participants in DPP were randomly assigned to receive an intensive lifestyle intervention (ILS), metformin (MET) or a placebo pill (PLB). ILS participants were offered an individualized 16-lesson curriculum over 24 weeks followed by monthly sessions through DPP. The curriculum focused on diet, exercise, and behavior change to a low-fat, low-calorie diet (<25% kcal from fat) and to perform ≥150 min/week of PA, with the primary goal to achieve ≥7% weight loss from baseline weight [17]. MET participants were assigned to take blinded 850g metformin twice daily; PLB participants were assigned a matching placebo pill twice daily. Both the MET and PLB groups received written standard lifestyle recommendations and a one-on-one lifestyle session annually [22]. Participants were followed for an average of 3.2 years.

Given the efficacy of ILS, DPP was terminated and participants’ groups were disclosed in July 2001; all participants were then offered the 16-session ILS curriculum [17] in group format through a Healthy Lifestyle Program (HELP) during a 6-month Bridge period [24] and invited to participate in the long-term follow-up study (DPPOS). DPPOS participants were offered group lifestyle HELP sessions every three months to reinforce weight and activity goals. ILS participants were offered an additional 2-4 booster lifestyle sessions twice annually. Metformin was continued unmasked in the MET group. Years of follow-up will be referred to as: years 0 (DPP baseline), 1 (DPP 1 year follow-up), and years 5, 6, 9, and 15 (DPPOS years 1, 2, 5, and 11). Protocols were approved by the local institutional review boards of participating study centers (Supplemental Table 1); all participants provided written informed consent.

### Exposure: The 2018 WCRF/AICR Score

The 2018 WCRF/AICR Score is used to estimate alignment with the 2018 WCRF/AICR Cancer Prevention Recommendations [6]. Eight recommendations operationalized within the standardized scoring system (Supplemental Table 2) address body weight, PA, fruit/vegetables and fiber, ultra-processed foods, red and processed meat, SSBs, and alcohol; the optional breastfeeding component was not included. Thus, total Scores ranged from 0-7 points, with a greater Score indicating greater alignment to the recommendations.

### Data collection

#### Body composition

The body weight component of the Score is calculated based on BMI (kg/m^2^) and WC. BMI was estimated from participants’ height (cm) and weight (kg). Height was attained at years 0, 1, and 15; the most recently measured height was used to calculate BMI at each visit. Weight was measured twice annually and WC (cm) was measured annually by trained personnel in duplicate. If there was a discrepancy larger than 0.5 cm for height and WC or 0.2 kg for weight, a third measure was taken and the average of the three were reported.

#### Physical activity

PA was collected at every annual visit through 15 years using the Modifiable Activity Questionnaire (MAQ), a valid and reliable tool to assess adult moderate and vigorous PA (MVPA) [25, 26]. The 37 activities included in the questionnaire were considered to be MVPA based on guidance from the 2011 Compendium of Physical Activities. As detailed in Supplemental Table 2, participants were categorized as meeting the PA recommendation if they performed ≥150 min/week of MVPA (equivalent of 7.5 MET hours/week).

#### Dietary intake

Study participants completed a modified version of the Insulin Resistance Atherosclerosis Study (IRAS) food frequency questionnaire (FFQ) in-person with trained personnel [27]. The 117-item questionnaire captured dietary recalls over the past year and was administered at years 0, 1, 5, 6, 9, and 15. Nutrient and energy estimates were calculated using the DietSys Nutrient Analysis Program and Nutrition Data System (version 2.6/8A/23, Nutrition Coordinating Center, University of Minnesota, Minneapolis, MN, USA) [27]. Data were used to calculate the five dietary components of the Score (fruits/vegetables and fiber, fast foods, red and processed meat, SSBs, and alcohol), as well as energy. Details of how each component was estimated are included in Supplemental Table 2.

#### Demographic covariates

Self-reported age (years), sex (male/female), race/ethnicity (Non-Hispanic White, Non-Hispanic Black, Hispanic, American Indian, Asian/Pacific Islander), education (years), smoking (never, current, former), family history of type 2 diabetes (yes/no), marital status (never married, living together, married, separated, divorced, widowed), and hormone therapy (in women, yes/no) were collected at year 0.

### Outcome

Fasting glucose and HbA1c were measured every six months and OGTTs were performed annually. The primary outcome was the development of diabetes based on the 1997 American Diabetes Association criteria: fasting plasma glucose ≥7 mmol/L (≥126 mg/dL) or 2-hour plasma glucose ≥11.1 mmol/L (≥200 mg/dL) after a 75g oral glucose load [21], confirmed by repeat test within six weeks. Participant outcomes were collected for DPP until July 31, 2001 and for DPPOS were used until January 2, 2014.

### Statistical analysis

Descriptive statistics were used to examine characteristics of the study population. Comparisons between groups were computed using ANOVA for continuous variables and chi-squared tests for categorical variables. Cox proportional hazard ratios (HRs) and 95% confidence intervals (CIs) were estimated for the association of the 2018 WCRF/AICR Score with incident diabetes over time, with person-years as the underlying time metric. The Score was modeled as a continuous variable (i.e., risk per 1-point increase). To examine if and how Scores changed over time (i.e., accounting for a time-dependent Score) and how they were associated with risk differences by group, the association between Score changes from years 0-1 on diabetes risk was examined through DPP (average 3 years follow-up) and through DPPOS (~15-years follow-up). Additionally, the association between time-dependent Score changes over 15 years and diabetes risk was examined. Treatment group, age, sex, race/ethnicity, and smoking were tested as potential effect modifiers; models were stratified as needed. Base models adjusted for age, sex, and baseline risk score. Multivariate models additionally adjusted for race/ethnicity, marital status, family history of type 2 diabetes, education, hormone therapy, and baseline energy intake.

Given the distribution of the data for fruit/vegetables and SSBs, sensitivity analyses were performed excluding participants with data outliers identified using the same approach described above to determine if they significantly affected estimates. Asian participant weight component cut-points were also adjusted in a second sensitivity analysis following World Health Organization (WHO) guidelines and WCRF/AICR Recommendations [6, 28]. Additionally, a sensitivity analysis was conducted to examine if findings differed by DPPOS lifestyle session attendance.

Lastly, models were run to explore the independent associations of each individual WCRF/AICR Score component. To further explore the effect of weight change and PA, models were run to assess associations with the body weight and PA components combined; the five nutrition components combined; and the Score excluding the weight component. All exploratory models adjusted for the other components in the Score and aforementioned covariates. SAS version 9.4 (SAS Institute, Inc., Cary, NC) was used for all analyses. Statistical tests were two-sided, with a significance level of 0.05.

## Results

### Study participant characteristics

Among the 3,147 participants, there were 611 cases of diabetes reported in DPP and 1,580 cases by year 15.

Mean baseline age of participants was 50.6 years; 68% were female (Table 1). Approximately half of participants were non-Hispanic White, 20% were non-Hispanic Black, and 16% were Hispanic; a small percent were Asian/Pacific Islander (5%) or American Indian (4.4%). Over half of participants were never smokers and only 7% were current smokers. Almost 70% of participants reported a family history of diabetes and 62% were married. The mean BMI was in the range of Class 1 obesity (33.9 kg/m^2^) and mean WC was 105.0 cm. There were no significant differences in participant characteristics or baseline Score by treatment group (Table 2).

**Table 1:** Baseline Characteristics of DPP and DPPOS participants by treatment group (*N* = 3,147)^a^

	All *N* = 3,147	Lifestyle *N* = 1,044	Metformin *N* = 1,044	Placebo *N* = 1,059
Age (years)	50.6 ± 10.7	50.6 ± 11.3	51.0 ± 10.3	50.3 ± 10.4
Female (%)	2131 (67.7)	711 (68.1)	692 (66.3)	728 (68.7)
Race/ethnicity (%)				
Non-Hispanic White	1741 (55.3)	572 (54.8)	592 (56.7)	577 (54.5)
Non-Hispanic Black	620 (19.7)	195 (18.7)	212 (20.3)	213 (20.1)
Hispanic	490 (15.6)	170 (16.3)	156 (14.9)	164 (15.5)
American Indian	157 (5.0)	53 (5.1)	48 (4.6)	56 (5.3)
Asian/Pacific Islander	139 (4.4)	54 (5.2)	36 (3.4)	49 (4.6)
Smoking Status (%)				
Never	1841 (58.5)	606 (58.0)	617 (59.1)	618 (58.4)
Former	1088 (34.6)	372 (35.6)	356 (34.1)	360 (34.0)
Current	218 (6.9)	66 (6.3)	71 (6.8)	81 (7.6)
Family History of Diabetes (%)	2182 (69.4)	727 (69.7)	709 (67.9)	746 (70.5)
Marital Status (%)				
Never Married	405 (12.9)	143 (13.7)	135 (12.9)	127 (12.0)
Living Together	121 (3.8)	44 (4.2)	32 (3.1)	45 (4.2)
Married	1954 (62.1)	644 (61.7)	646 (61.9)	664 (62.7)
Separated	88 (2.8)	30 (2.9)	31 (3.0)	27 (2.5)
Divorced	437 (13.9)	134 (12.8)	154 (14.8)	149 (14.1)
Widowed	142 (4.5)	49 (4.7)	46 (4.4)	47 (4.4)
Education (years)	14.8 ± 3.1	14.8 ± 3.1	14.9 ± 3.0	14.8 ± 3.2
Receiving hormone therapy (female) (%)	528 (16.8)	183 (17.5)	191 (18.3)	154 (14.5)
BMI (kg/m^2^)	33.9 ± 6.6	33.8 ± 6.7	33.8 ± 6.6	34.1 ± 6.6
Waist (cm)	105.0 ± 14.4	105.0 ± 14.7	104.8 ± 14.4	105.2 ± 14.2
Fasting glucose (mg/dl)	106.5 ± 8.3	106.2 ± 8.0	106.5 ± 8.5	106.7 ± 8.4
2-Hour glucose (mg/dl)	164.6 ± 17.0	164.3 ± 16.8	165.0 ± 17.3	164.6 ± 17.1
HbA1c (%)	5.9 ± 0.5	5.9 ± 0.5	5.9 ± 0.5	5.9 ± 0.5
Total energy intake (kcal)^b^	1891 (1453, 2540)	1874 (1451, 2533)	1914 (1474, 2606)	1889 (1424, 2506)
Leisure MET (hours)^b^	9.9 (4.0, 20.6)	9.7 (4.0, 19.6)	10.1 (4.0, 20.7)	10.0 (3.9, 21.2)
Fruits and Vegetables (serv/day) ^b^	3.8 (2.4, 5.6)	3.7 (2.5, 5.7)	3.8 (2.4, 5.5)	3.8 (2.3, 5.6)
Fiber (g/day) ^b^	14.8 (10.6, 20.1)	14.9 (10.6, 20.3)	14.9 (10.7, 20.0)	14.5 (10.2, 19.9)
Sugar-sweetened beverages (g/day) ^b^	96.3 (11.3, 266.3)	89.4 (16.9, 257.5)	107.5 (17.5, 69.4]	90.0 (8.8, 253.8)
Alcohol (drinks/day) ^b^	0.0 (0.0, 0.1)	0.0 (0.0, 0.1)	0.0 (0.0, 0.2)	0.0 (0.0 , 0.1)
Red meat (g/day) ^b^	241.0 (110.1, 428.4)	238.0 (107.1, 422.5)	249.9 (119.0, 441.8)	238.0 (101.2, 422.5)
Processed meat (g/day) ^b^	122.0 (44.6, 276.7)	130.9 (47.6, 281.1)	125.0 (46.1, 285.6)	116.0 (41.7, 258.8)
UPFs (% kcal/day)	0.4 ± 0.1	0.4 ± 0.1	0.4 ± 0.1	0.4 ± 0.1

*BMI* body mass index, *DPP* Diabetes Prevention Program, *DPPOS* Diabetes Prevention Program Outcome Study, *kcal* kilocalories, *MET* metabolic equivalent of task, *serv* servings, *UPFs* ultra-processed foods

^a^Data are N (%) or mean±SD unless otherwise noted. P-values reported are for ANOVA for continuous variables and χ^2^ tests for categorical variables. There were no significant differences by treatment group (*p*>0.05).

^b^Data are median (25^th^ and 75^th^ percentile). *P*-values reported are for Wilcoxon rank sum test. There were no significant differences by treatment group (*p*>0.05).

**Table 2:** Participant alignment with the 2018 WCRF/AICR Score- total Score and by component (*N*=3,147)^a^

Baseline Score and Components	Lifestyle *N = 1044*	Metformin *N = 1044*	Placebo *N = 1059*
Total WCRF/AICR Score	3.24 ± 1.10	3.22 ± 1.06	3.25 ± 1.08
By component *(N (%))*			
Body weight			
Did not meet	665 (63.7)	675 (64.7)	688 (65.0)
Partially Met	361 (34.6)	351 (33.6)	358 (33.8)
Met	18 (1.7)	18 (1.7)	13 (1.2)
Physical activity			
Did not meet	246 (23.6)	246 (23.6)	258 (24.4)
Partially Met	184 (17.6)	184 (17.6)	176 (16.6)
Met	614 (58.8)	614 (58.8)	625 (59.0)
Plant-based foods			
Did not meet	228 (21.8)	253 (24.2)	269 (25.4)
Partially Met	759 (72.7)	723 (69.3)	722 (68.2)
Met	57 (5.5)	68 (6.5)	68 (6.4)
Fast-foods			
Did not meet	351 (33.6)	336 (32.2)	358 (33.8)
Partially Met	354 (33.9)	353 (33.8)	346 (32.7)
Met	339 (32.5)	355 (34.0)	355 (33.5)
Red and processed meat			
Did not meet	615 (58.9)	629 (60.2)	621 (58.6)
Partially Met	256 (24.5)	245 (23.5)	248 (23.4)
Met	173 (16.6)	170 (16.3)	190 (17.9)
Sugar-sweetened beverages			
Did not meet	267 (25.6)	276 (26.4)	266 (25.1)
Partially Met	564 (54.0)	551 (52.8)	559 (52.8)
Met	213 (20.4)	217 (20.8)	234 (22.1)
Alcohol			
Did not meet	30 (2.9)	38 (3.6)	26 (2.5)
Partially Met	490 (46.9)	493 (47.2)	494 (46.6)
Met	524 (50.2)	513 (49.1)	539 (50.9)

*AICR* American Institute for Cancer Research, *WCRF* World Cancer Research Fund

^a^Data are N (%). *P*-value for χ^2^ tests across treatment groups. There were no significant differences by treatment group (*p*>0.05).

Continuous distributions of Score components are included in Table 1. Table 2 includes a breakdown of the proportion of participants meeting, partially meeting, or not meeting each of the Score’s recommendations at baseline. The mean WCRF/AICR Score across groups was 3.24 out of 7 points; the majority of participants did not meet body weight, PA, and red and processed meat recommendations. However, most participants met or partially met the recommendations for plant-based foods, SSBs, and alcohol. There were no significant differences by treatment group (Table 2).

**Fig. 1:**
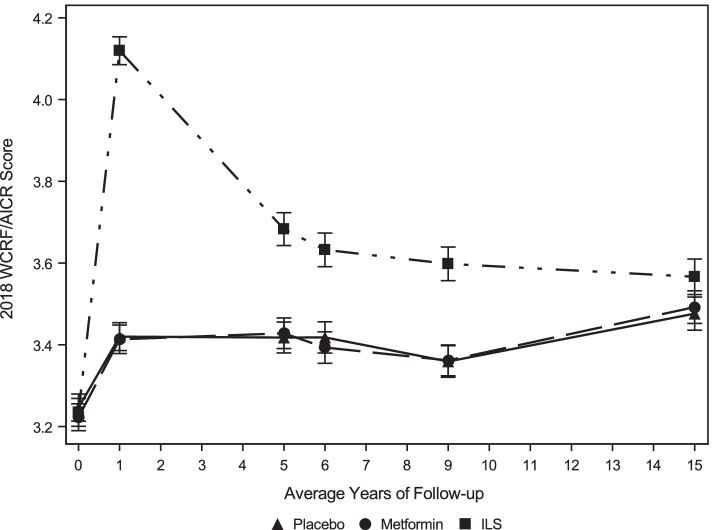
Changes in DPP and DPPOS participants 2018 WCRF/AICR Scores over time, by treatment group^1^. AICR, American Institute for Cancer Research; DPP, Diabetes Prevention Program; DPPOS, Diabetes Prevention Program Outcomes Study; WCRF, World Cancer Research Fund. ^1^Data shown are mean±SEM. The results are unadjusted for covariates. 2018 WCRF/AICR Scores were calculated at years 0, 1, 5, 6, 9, and 15 by the three DPP treatment groups. Triangles = placebo group, circles = Metformin group, squares = intensive lifestyle group

There were Score improvements within all treatment groups from years 0-1, as well as at year 15 (*p* < 0.0001, Fig. 1). When comparing Scores between groups, the ILS group had greater Score improvements compared to the MET and PLB groups between years 0-1 (*p* < 0.001), however, there were no significant differences between groups at year 15 (*p* = 0.237). Asian/Pacific Islander participants consistently had the highest Scores, followed by Hispanic and Non-Hispanic White participants. Non-Hispanic Black and American Indian participants consistently had the lowest Scores over 15 years (Supplemental Figure 2).

#### 2018 WCRF/AICR Score changes over time and diabetes risk

There was a significant interaction between both treatment group race/ethnicity and change in Score from years 0-1 through the end of DPP on diabetes risk (*p*=0.032 and 0.044, respectively), but not through year 15 or when examining time-dependent Score changes and risk. There were no significant interactions between age, sex, or smoking status (p-interaction >0.05) and Score on diabetes risk. For every one-unit improvement in Score from years 0-1, those in the ILS and PLB groups had a 31% (HR: 0.69, 95% CI: 0.56-0.84) and 15% (HR: 0.85, 95% CI: 0.73-0.98) reduction in diabetes risk through the end of DPP, respectively. Findings were not significant for the MET group (HR: 0.95, 95% CI: 0.79-1.14) (Table 3). Additionally, when stratified by race/ethnicity, associations were strongest among American Indian participants (HR: 0.33 (95% CI: 0.17-0.66), followed by Non-Hispanic White (HR: 0.75, 95% CI: 0.66-0.85) and Hispanic participants (HR: 0.79, 95% CI: 0.63-0.99); findings were not significant in non-Hispanic Black and Asian/Pacific Islander participants (Supplemental Table 3).

**Table 3:** Hazard ratios and 95% confidence intervals for improvement in the 2018 WCRF/AICR Score and diabetes risk by treatment group^a,b^

	Overall	Lifestyle	Metformin	Placebo
Score change between years 0 and 1 and risk through end of DPP^c^				
*Cases/N*	*611/2900*	*138/961*	*196/968*	*277/971*
Base^d^	**0.83 (0.75, 0.91)**	**0.69 (0.56, 0.84)**	0.95 (0.79, 1.14)	**0.84 (0.72, 0.97)**
Multivariate^e^	**0.83 (0.75, 0.91)**	**0.69 (0.56, 0.84)**	0.95 (0.79, 1.14)	**0.85 (0.73, 0.98)**
Score change between year 0 and 1 and risk through year 15^f^				
*Cases/N*	*1490/2900*	*463/961*	*481/968*	*546/971*
Base^d^	**0.87 (0.81, 0.92)**	**0.80 (0.71, 0.89)**	0.95 (0.85, 1.06)	**0.86 (0.78, 0.96)**
Multivariate^e^	**0.87 (0.82, 0.93)**	**0.81 (0.72, 0.90)**	0.95 (0.85, 1.06)	**0.87 (0.78, 0.96)**
Time-dependent Score (years 0 to 15) and risk through year 15^f^				
*Cases/N*	*1580/3147*	*486/1044*	*515/1044*	*579/1059*
Base^d^	**0.87 (0.82, 0.93)**	**0.82 (0.73, 0.91)**	0.95 (0.85, 1.06)	**0.85 (0.77, 0.94)**
Multivariate^e^	**0.88 (0.83, 0.93)**	**0.82 (0.74, 0.92)**	0.95 (0.85, 1.06)	**0.85 (0.77, 0.94)**

*AICR* American Institute for Cancer Research, *DPP* Diabetes Prevention Program, *WCRF* World Cancer Research Fund. Results with significant *p*-values (*p*<0.05) are bolded. Data shown are HR for incident diabetes per one-point positive change in Score

^a^The N included examining Score change between years 0 and 1 is slightly lower than the time-dependent Score analysis because some participants were missing year 1 nutrition data but have subsequent years’ nutrition data.

^b^Diabetes is defined based on the 1997 American Diabetes Association criteria: fasting plasma glucose ≥ 7 mmol/L (≥ 126 mg/dL) measured every six months or 2-hour plasma glucose ≥ 11.1 mmol/L (≥200 mg/dL) after a 75 g oral glucose load [21].

^c^Year 0 through the end of the Diabetes Prevention Program represents a mean follow-up time of 3.2 years

^d^Base model adjusted for age, sex, and baseline risk score

^e^Multivariate model adjusted age, sex, race/ethnicity, marital status, family history of type 2 diabetes, smoking status, years of education, hormone therapy, baseline total caloric intake, and baseline risk score

^f^DPPOS follow-up through January 2014 was included in the study, representing a mean follow-up time of 15 years

Overall, every one-point improvement in 2018 WCRF/ACIR Scores between years 0-1 was associated with a 13% reduction in diabetes incidence (95% CI: 0.82-0.93) through year 15 (Table 3). When examined by treatment group, findings were only significant in the ILS (HR: 0.81, 95% CI: 0.72-0.90) and PLB groups (HR: 0.87, 95% CI: 0.78-0.96). When the time-dependent Score was examined over 15 years, there was a similar reduction in risk per one-unit change overall (HR: 0.88, 95% CI 0.83-0.93) and in the ILS (HR: 0.82, 95% CI 0.74-0.92) and PLB groups (HR: 0.85, 95% CI: 0.77-0.94), but not the MET group (Table 3).

#### Sensitivity analysis

Excluding seven participants (*N*=3,140) with outliers for fruit and vegetables and SSBs did not affect results (data not shown). Asian/Pacific Islander participants’ Scores decreased when the body weight component cut-points were adjusted (Supplemental Figure 3), but the weight component did not fully account for the difference in Scores compared to other race/ethnic groups; they continued to have the highest Scores over time.

Lifestyle class attendance during DPPOS was low across intervention groups: approximately 80% of participants attended fewer than a quarter of the sessions offered. Moreover, a similar pattern of Score change was found over time regardless of DPPOS class attendance rate (data not shown).

#### 2018 WCRF/AICR Score components change over time and diabetes risk

Results for the association between change in the seven components of the 2018 WCRF/AICR Score and diabetes risk are included in Supplemental Table 4. Alignment with the body weight recommendation was associated with the greatest reduction in diabetes risk across treatment groups over time, although associations only remained significant across comparisons for the ILS group. Alignment with PA and plant-based diet recommendations were associated with the next greatest reduction in diabetes risk, though this was not consistent across groups. Additionally, the red and processed meat component was significantly associated with diabetes risk in the PLB group over 15-years follow-up (Supplemental Table 4).

The association between changes in weight and PA components combined, five nutrition components combined, and total Score excluding the weight component and diabetes risk are included in Supplemental Table 5. Changes in alignment with weight and PA components combined after year 1 and over time were associated with significant reductions in diabetes risk in the ILS group; risk decreased for all groups when time-dependent Scores were examined, though the greatest risk reductions remained in the ILS group. Alignment with the five nutrition components combined was associated with reduced diabetes risk in the PLB group only when looking at Score changes from years 0-1 and risk through year 15. Scores excluding the weight component were only significantly associated with reduced diabetes risk in the PLB group.

## Discussion

The current study examined if following a lifestyle aligned with the 2018 WCRF/AICR Cancer Prevention Recommendations significantly lowers risk for diabetes in adults at high risk of developing diabetes. The hypotheses were that improving alignment with the guidelines via improved 2018 WCRF/AICR Scores would be associated with lower diabetes risk over time, and that the association between the Score and risk would differ by DPP intervention group. Specifically, we anticipated the greatest improvements in Scores and reduction in diabetes risk in the ILS group and least changes in the PLB group. When examining the association between changes in Scores from years 0-1 and diabetes incidence through the end of DPP and DPPOS, as well as the association between time-dependent Score changes and risk through DPPOS, those in the ILS group had the greatest overall reduction in risk. However, those in the PLB group also had a reduced risk of diabetes with improving Score through DPP and DPPOS; findings in the MET group were not significant.

These findings add to our understanding of how lifestyle recommendations for cancer prevention may impact diabetes risk. The greatest improvements in Scores were seen between years 0-1, particularly for the ILS group. This is not unexpected, given it was during the intensive DPP intervention period; over the next 11 years, as support through the program decreased, ILS Scores slowly approached those of the MET and PLB groups. In the PLB group, there was also a significant reduction in diabetes risk with increasing Score. Over the course of the Bridge period and DPPOS, one could postulate that identification of being in the PLB group may have prompted participant efforts to improve lifestyle behaviors. However, fewer than six lifestyle sessions were attended on average by PLB participants, and PLB Scores did not significantly improve in DPPOS compared to MET or ILS. Another explanation may be the presence of survivorship bias, where those who are healthier remain in the cohort and thus appear to have a lower risk of diabetes. This may also explain the increase in Scores across groups from years 9-15. Finally, the MET group did not have a risk reduction in relation to the Score compared to the other groups; taking metformin may have had the strongest impact on the overall diabetes risk of these participants. Aside from suppression of hepatic gluconeogenesis, metformin may reduce diabetes risk through appetite and caloric intake rather than energy expenditure, thus minimizing the impact of the Score’s dietary and PA components.

Another finding of this study was that Asian/Pacific Islander participants consistently had the highest Scores over 15 years, including after body weight component cut-points were adjusted, followed by Hispanic and Non-Hispanic White participants; non-Hispanic Black and American Indian participants consistently had the lowest Scores. However, when stratified models were examined for Score changes from years 0-1 and associated risk through DPP, the greatest inverse associations were seen among American Indian participants, followed by Non-Hispanic White and Hispanic participants; findings were not significant among Asian/Pacific Islander and non-Hispanic Black participants. Underlying biological differences in glucose or insulin metabolism [29, 30] and genetic factors may differentially impact risk for diabetes [31, 32] as well as other factors, including socioeconomic factors and differences in cultural eating patterns [32]. Stratified findings may also have been affected, though, by the broadness of race/ethnic categories – i.e., the capturing of genetic and cultural heterogeneity within each group [31] – and/or the limited sample size for each group. Future studies in diverse populations may help elucidate these differences in associations by race/ethnicity.

To the best of our knowledge, this is the first study to examine the association between WCRF/AICR Cancer Prevention Recommendations and diabetes risk. The Recommendations encompass not just weight loss and PA, but also diet quality goals. Past studies in DPP/DPPOS examined the association of individual lifestyle factors and diabetes risk. Such studies in DPP observed that improvements in diet quality as well as changes in macronutrient consumption (e.g., higher high-fiber carbohydrate intake and lower total and saturated fat intake) over one year predicted weight loss but did not predict reductions in diabetes incidence [33, 34]. Hamman et al. examined the impact of meeting ILS goals among the ILS group and found that neither meeting PA or fat gram goals predicted diabetes incidence once adjusting for weight loss [18]. When extended to DPPOS, Kriska et al. found that PA was related to a reduction in weight and diabetes incidence over an average 12 years of follow-up for the overall cohort and most significantly in the ILS group [35]. Together these studies on individual lifestyle factors support our findings suggesting that improvements in a combination of lifestyle behaviors can positively impact diabetes risk.

Our exploratory by-component analysis suggests that change in body weight is a main driver of the Score’s association with diabetes risk. This is expected given that the DPP study enrolled participants with overweight and obesity and there is strong evidence that weight loss is a major contributor to reduced diabetes incidence [18, 36]. However, exploratory findings suggest PA, plant-based foods, and fast foods components may also be driving some of the associations seen in this study. Both the five nutrition components combined, as well as the Score excluding the weight component, were also associated with reduced diabetes risk in the PLB group.

Additionally, given this study had a small proportion of smokers and overall low alcohol consumption, it is unsurprising there was no effect modification by smoking status or that, overall, alcohol was not a driving factor in this population. Though there was an increased risk of diabetes with greater alignment with the alcohol recommendation in the ILS group, this finding was consistent with previous research, which has reported that moderate alcohol use is associated with lower diabetes risk and lower insulin secretion at similar levels of insulin resistance [37, 38]. The relationship observed in this study may be due to low rates of alcohol consumption in the cohort or may be confounded by other unmeasured factors; however, despite this paradoxical association, the association between higher Scores and decreased diabetes incidence was still robust. Indeed, the Score was not developed to investigate one component alone, and adherence to each recommendation and the impact of each component on disease risk may vary by population. Many 2018 WCRF/AICR Cancer Prevention Recommendations included in the Score are also interrelated (e.g., fast food and SSB recommendations are based on evidence linking intake with obesity). Greater emphasis should thus be on the total Score and examining how the combined recommendations impact disease risk.

There are many strengths in this study. First, this study used a standardized scoring system for lifestyle behavior changes. Second, although the ILS curriculum in DPP did not specifically examine adherence to the 2018 WCRF/AICR Cancer Prevention Recommendations, their guidance on body weight, PA goals, and healthy eating aligned well with them. Additionally, the lifestyle sessions that reinforced the ILS group’s healthy choices and the lifestyle sessions provided to all participants after DPP provided an opportunity to further examine how changes in compliance impacted diabetes risk. Lastly, the study included a diverse group of participants, data were collected at multiple time points allowing for the study of time-dependent Score changes, and the follow-up period beyond the DPP intervention enabled examination of diabetes risk over 15 years.

Limitations included recall bias and potential misclassification due to measurement error from self-report questionnaires. The cut-points in the WCRF/AICR Score may also not be ideal for all participants. E.g., WHO and the 2018 WCRF/AICR 3^rd^ expert report suggest alternate BMI and WC cut-points for Asian adults. However, in a sensitivity analysis with updated cut-points, Asian/Pacific Islander participants remained the highest scoring group. Additionally, though this study was in a diverse sample of participants, there were small sample sizes for some race/ethnic groups in stratified analyses. Future studies can examine if there are similar differences in associations by race/ethnicity.

As previously mentioned, there is also the potential for survivor bias. Lastly, all components are weighted equally, though there are likely differential effects. Future methodological work could examine the implications of reweighting components.

## Conclusions

Our study suggests that adapting a lifestyle to better align with the 2018 WCRF/AICR Cancer Prevention Recommendations may reduce risk of diabetes in high-risk adults with pre-diabetes. Weight loss alone did not appear to be the only driver of reduced risk for diabetes, but also PA and dietary changes. Future research should explore how diabetes risk reduction may impact cancer risk in populations with prediabetes.

## Supplementary Information

**Additional file 1.** Supplemental Tables and Figures.

## Data Availability

The datasets used and/or analyzed during the current study are available from the corresponding author on reasonable request. In accordance with the NIH Public Access Policy, we continue to provide all manuscripts to PubMed Central including this manuscript DPP/DPPOS has provided the protocols and lifestyle and medication intervention manuals to the public through its public website (https://www.dppos.org). The DPPOS abides by the NIDDK data sharing policy and implementation guidance as required by the NIH/NIDDK (https://www.niddkrepository.org/studies/dppos/). All data are available through the NIDDK Data Repository (https://repository.niddk.nih.gov/studies/dppos/).

## Abbreviations

AICR: American Institute for Cancer Research
BMI: Body mass index
CI: Confidence interval
DPP: Diabetes Prevention Program
DPPOS: Diabetes Prevention Program Outcomes study
FFQ: Food frequency questionnaire
HR: Hazard ratios
ILS: Intensive lifestyle intervention
MET: Metformin
MVPA: Moderate and vigorous PA
NCD: Non-communicable disease
OGTT: Oral glucose tolerance test
PA: Physical activity
PLB: Placebo pill
SSB: Sugar-sweetened beverage
WC: Waist circumference
WCRF: World Cancer Research Fund
WHO: World Health Organization
