# Supplementary material for: The 2018 World Cancer Research Fund (WCRF)/American Institute for Cancer Research (AICR) score and diabetes risk in the Diabetes Prevention Program Outcomes Study (DPPOS)
Source: BMC Nutr. 2022 Sep 21;8:105. doi: 10.1186/s40795-022-00596-7 (PMC9494851; doi:10.1186/s40795-022-00596-7)
Supplement: Supplementary file 1 — Additional file 1. Supplemental Tables and Figures. [file 40795_2022_596_MOESM1_ESM.docx]

**The 2018 World Cancer Research Fund (WCRF)/American Institute for Cancer Research (AICR) Score and Diabetes Risk in the Diabetes Prevention Program Outcomes Study (DPPOS)**

Marissa M. Shams-White, PhD; Ashley H. Tjaden, MPH; Sharon L. Edelstein, ScM; Sarah Bassiouni, MPH; Lisa L. Kahle, BA; Catherine Kim, MD; Xavier Pi-Sunyer, MD; Karla A. Temple, PhD; Elizabeth M. Venditti, PhD; Jill Reedy, PhD; Brandy M. Heckman- Stoddard, PhD; DPP Research Group

Contents:

1. **Supplemental Figure 1.** Flow diagram of included DPP/DPPOS participants
2. **Supplemental Table 1.** Institutional IRBs for Approval of DPP
3. **Supplemental Table 2.** 2018 WCRF/AICR Scoring breakdown system
4. **Supplemental Figure 2**. Changes in DPP and DPPOS participants’ 2018 WCRF/AICR Scores over time, by race/ethnicity
5. **Supplemental Table 2.** Hazard ratios and 95% confidence intervals for improvement in the 2018 WCRF/AICR Score and diabetes risk by race/ethnicity
6. **Supplemental Figure 3**. Changes in DPP and DPPOS participants’ 2018 WCRF/AICR Scores over time, by race/ethnicity, adjusting weight component cut-points for Asian/Pacific Islander participants
7. **Supplemental Table 4**. Hazard ratios and 95% confidence intervals for change in the seven components of the 2018 WCRF/AICR Score (0-7 points) and diabetes risk by treatment group
8. **Supplemental Table 5**. Hazard ratios and 95% confidence intervals for change in the components of the 2018 WCRF/AICR Score (0-7 points) and diabetes risk by treatment group

**Supplemental Figure 1.** Flow diagram of included DPP/DPPOS participants

**Supplemental Table 1**. Institutional IRBs for Approval of DPP

| Institution | City | State | Name of IRB |
| --- | --- | --- | --- |
| Pennington Biomedical Research Center | Baton Rouge | LA | Pennington Biomedical Research Center IRB |
| University of Chicago | Chicago | IL | University of Chicago IRB |
| Jefferson Medical College | Philadelphia | PA | Thomas Jefferson University IRB |
| University of Miami | Miami | FL | University of Miami IRB |
| University of Texas Health Science Center at San Antonio | San Antonio | TX | UT Health IRB |
| University of Colorado | Denver | CO | Colorado Multiple Institutional Review Board |
| Joslin Diabetes Center | Boston | MA | Committee On Human Studies Joslin Diabetes Center |
| University of Washington | Seattle | WA | University of Washington IRB/Veterans Affairs IRB |
| University of Tennessee | Memphis | TN | University Of Tennessee Health Science IRB |
| Northwestern University | Chicago | IL | Northwestern University IRB |
| Massachusetts General Hospital | Boston | MA | Massachusetts General Hospital/Partners Human Research Committee |
| University of California, San Diego | San Diego | CA | UCSD Human Research Protections Program |
| Columbia University (formerly St. Luke's-Roosevelt Hospital Center) | New York | NY | St. Luke's-Roosevelt Hospital Center IRB/Human Research Protection Office CUMC IRB |
| Indiana University | Indianapolis | IN | Indiana University |
| Medstar Research Institute | Hyattsville | MD | Georgetown - Medstar IRB System |
| University of California, Los Angeles | Alhambra | CA | UCLA Medical IRB1 |
| Washington University School of Medicine | St Louis | MO | Washington University Institutional Review Board |
| Johns Hopkins School of Medicine | Baltimore | MD | Johns Hopkins Medicine IRB1 |
| The University of New Mexico | Albuquerque | NM | UNM HSC Human Research Review Committee |
| Albert Einstein College of Medicine | Bronx | NY | Albert Einstein College of Medicine |
| University of Pittsburgh | Pittsburgh | PA | University of Pittsburgh IRB |
| University of Hawaii | Honolulu | HI | University of Hawaii Human Studies Program |
| SW Indian Center – Salt River/Phoenix | Phoenix | AZ | National Institutes of Health Intramural Institutional Review Board |
| SW Indian Center - Zuni | Zuni | NM | National Institutes of Health Intramural Institutional Review Board |
| SW Indian Center - Gila River | Gila River | AZ | National Institutes of Health Intramural Institutional Review Board |
| SW Indian Center - Shiprock | Shiprock | NM | National Institutes of Health Intramural Institutional Review Board |
| University of Southern California | Los Angeles | CA | USC Office for the Protection of Research Subjects |

### **Supplemental Table 2.** 2018 WCRF/AICR Scoring breakdown system

| **Score components** | **Operationalization of Recommendations** | | **Meeting Recommendation Point System** |
| --- | --- | --- | --- |
|  | **Recommended scoring** | **Scoring adaptations^1^** |  |
| 1. **Body weight** | **Body mass index** (kg/m^2^): | *None* |  |
|  | 18.5-24.9 | --- | Met 0.5 |
|  | 25-29.9 | --- | Partially 0.25 |
|  | <18.5 or ≥30 | --- | Not Met 0 |
|  | **Waist circumference** (inches): |  |  |
|  | M: <37 F: <31.5 | --- | Met 0.5 |
|  | M: 37-<40 F: 31.5-<35 | --- | Partially 0.25 |
|  | M: ≥40 F: ≥35 | --- | Not Met 0 |
| 1. **Physical Activity** | **Total MVPA** (minutes/week): | (MET-hours/week) |  |
|  | ≥150 | ≥7.5 | Met 1.0 |
|  | 75-<150 | 3.75-7.5 | Partially 0.5 |
|  | <75 | <3.75 | Not Met 0 |
| 1. **Plant-based foods** | **Fruits and vegetables** (g/day) | (servings/day)^2^ |  |
|  | ≥ 400 | ≥ 5 | Met 0.5 |
|  | 200- <400 | 2.5 - <5 | Partially 0.25 |
|  | <200 | <2.5 | Not Met 0 |
|  | **Total Fiber** (g/day): | *None* |  |
|  | ≥ 30 | --- | Met 0.5 |
|  | 15-<30 | --- | Partially 0.25 |
|  | <15 | --- | Not Met 0 |
| 1. **Fast foods** | **% total kcal from ultra-processed foods:** | *None*^3^ |  |
|  | Tertile 1 | --- | Met 1.0 |
|  | Tertile 2 | --- | Partially 0.5 |
|  | Tertile 3 | --- | Not Met 0 |
| 1. **Red and processed meat** | **Total red & processed meat** (g/week): | *None*^4^ |  |
|  | Red ≤500 and processed <21 | --- | Met 1.0 |
|  | Red ≤500 and processed 21-<100 | --- | Partially 0.5 |
|  | Red >500 or processed ≥100 | --- | Not Met 0 |
| 1. **Sugar-sweetened beverages** | **Total sugar-sweetened beverages** (g/day): | (servings/day)^5^ |  |
|  | 0 | 0 | Met 1.0 |
|  | >0-≤ 250 | >0 - ≤1 | Partially 0.5 |
|  | >250 | >1 | Not Met 0 |
| 1. **Alcohol** | **Total alcohol** (drinks/day): | *None* |  |
|  | 0 | --- | Met 1.0 |
|  | M: >0-≤2 F: >0-≤1 | --- | Partially 0.5 |
|  | M: >2 F: >1 | --- | Not Met 0 |
| **Total Score Range** | |  | 0-7 |

AICR, American Institute for Cancer Research; DPP, Diabetes Prevention Program; DPPOS, Diabetes Prevention Program Outcomes Study WCRF, World Cancer Research Fund F, females; M, males, MET, metabolic equivalent of task

^1^ Adaptations made to the scoring system due to differences in units included in the dataset are listed where applicable.

^2^ Fruit and vegetables were available in the dataset as servings/day. Given that the 2018 WCRF/AICR Third Expert Report states five portions or servings is equivalent to 400 grams, the cut-points were converted from grams to servings per day.

^3^ To estimate percent of total kcal from ultra-processed foods, the kilocalories per questionnaire line item were calculated using data from the University of Minnesota’s Nutrition Coordinating Center. Given that open food codes could not be re-estimated per individual line item, they were excluded from the aUPF list and total kcal were re-estimated to better estimate percent of total calories from aUPF. Any items already included in the other components (e.g., red or processed meat, sugar-sweetened beverages) were excluded.

^4^ Red and processed meat were available in the dataset as servings/day. They were converted to grams/week, assuming 1 serving = 85 grams (i.e., [number of servings/day x 85g] x 7 days/week = grams/week)

^5^ Sugar-sweetened beverages were available in the dataset as servings/day. SSBs include “Regular soft drinks (including colas, 7-up, etc.,” “lemonade, sweetened mineral water,” and “Other fruit juice with vitamin C, fortified fruit drinks, Hi-C, Kool-Aid, cranberry juice, tang.” 100% fruit juice was not included. They were converted to grams/week, assuming 1 serving = 250 grams (i.e., number of servings/day x 250 grams = grams/day).


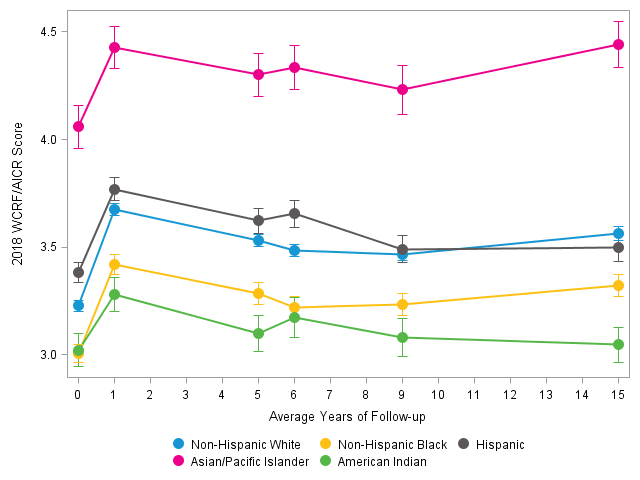


**Supplemental Figure 2.** Changes in DPP and DPPOS participants’ 2018 WCRF/AICR Scores over time, by race/ethnicity^1^

Data shown for all participants combined.

^1^ The results are unadjusted for covariates. 2018 WCRF/AICR Scores were calculated at years 0, 1, 5, 6, 9, and 15.

## **Supplemental Table 3.** Hazard ratios and 95% confidence intervals for improvement in the 2018 WCRF/AICR Score and diabetes risk by race/ethnicity^1,2^

| **Score change between years 0 and 1 and risk through end of DPP**^3^ | | | | | | |
| --- | --- | --- | --- | --- | --- | --- |
|  | **Overall** | **Non-Hispanic White** | **Non-Hispanic**  **Black** | **Hispanic** | **Asian & Pacific Islander** | **American Indian** |
| *Cases/N* | *611/2900* | *333/1607* | *120/560* | *99/456* | *29/122* | *30/153* |
| Multivariate^5^ | **0.83 (0.75, 0.91)** | **0.75 (0.66, 0.85)** | 0.82 (0.67, 1.01) | **0.79 (0.63, 0.99)** | 0.63 (0.39, 1.04) | **0.33 (0.17, 0.66)** |

AICR, American Institute for Cancer Research; DPP, Diabetes Prevention Program; WCRF, World Cancer Research Fund. Results with significant p-values (p<0.05) are bolded. Data shown are hazard ratios for incident diabetes per one-point positive change in Score.

^1^ The N included examining Score change between years 0 and 1 is slightly lower than the time-dependent Score analysis because some participants were missing year 1 nutrition data but have subsequent years’ nutrition data.

^2^ Diabetes is defined based on the 1997 American Diabetes Association criteria: fasting plasma glucose ≥ 7 mmol/L (≥ 126 mg/dL) measured every six months or 2-hour plasma glucose ≥ 11.1 mmol/L (≥200 mg/dL) after a 75 g oral glucose load (21).

^3^ Year 0 through the end of the Diabetes Prevention Program represents a mean follow-up time of 3.2 years

^4^ There was no significant effect modification by race/ethnicity in the base model, which adjusted for age, sex, and baseline risk score (p=0.058). There was significant effect modification between Score changes and race/ethnicity in the multivariate model (p=0.044).Multivariate model adjusted age, sex, race/ethnicity, marital status, family history of type 2 diabetes, smoking status, years of education, hormone therapy, baseline total caloric intake, and baseline risk score.


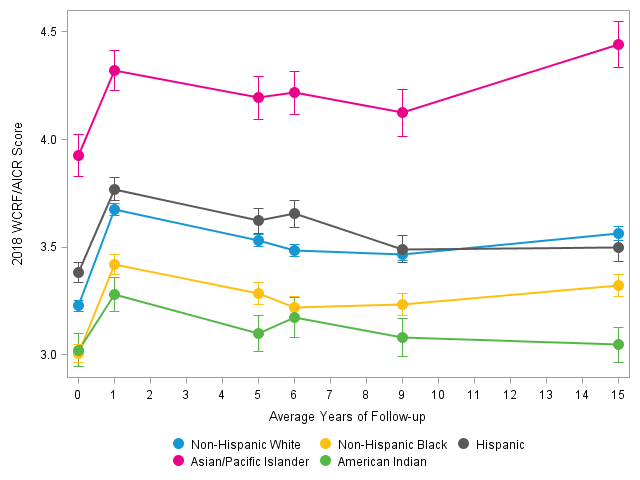


**Supplemental Figure 3.** Changes in DPP and DPPOS participants’ 2018 WCRF/AICR Scores over time, by race/ethnicity, adjusting weight component cut-points for Asian/Pacific Islander participants^1,2^

Data shown for all participants combined.

^1^ The results are unadjusted for covariates. 2018 WCRF/AICR Scores were calculated at years 0, 1, 5, 6, 9, and 15.

^2^Asian participant cut-points for BMI and waist circumference based on WHO guidelines and WCRF/AICR recommendations. Compared to other participants BMI cut-points changed for 1 point: 18.5-22.9 (versus 18.5-24.9); 0.5 points: 23-27.5 (versus 25-29.9), and 0 points: <18.5 or ≥27.5 (versus <18.5 and ≥30). For waist circumference, only male cut point values lowered, where 1 point: <90 cm (versus <94 cm), 0.5 points: 90 - <102 cm (versus 94 - <102cm for others), and 0 points remained ≥102 cm.

## **Supplemental Table 4.** Hazard ratios and 95% confidence intervals for change in the seven components of the 2018 WCRF/AICR Score (0-7 points) and diabetes risk by treatment group^1,2^

|  | **Score change between years 0 and 1 and risk through end of DPP**^3,4^ | | | **Score change between year 0 and 1 and risk through year 15**^4^**^,^**^5^ | | | **Time-dependent Score (years 0 to 15) on risk through year 15**^5,6^ | | |
| --- | --- | --- | --- | --- | --- | --- | --- | --- | --- |
| **Component** | **Lifestyle** | **Metformin** | **Placebo** | **Lifestyle** | **Metformin** | **Placebo** | **Lifestyle** | **Metformin** | **Placebo** |
| *Cases/N* | *138/961* | *196/968* | *277/971* | *463/961* | *481/968* | *546/971* | *486/1044* | *515/1044* | *579/1059* |
| Body Weight | **0.04 (0.01, 0.12)** | 0.49 (0.18, 1.31) | 0.79 (0.33, 1.91) | **0.20 (0.12, 0.34)** | **0.38 (0.20, 0.72)** | 1.10 (0.98, 1.04) | **0.32 (0.23, 0.44)** | **0.55 (0.38, 0.78)** | **0.53 (0.37, 0.77)** |
| Physical Activity | **0.50 (0.31, 0.80)** | 1.26 (0.87, 1.82) | 0.84 (0.63, 1.13) | **0.70 (0.54, 0.91)** | 1.00 (0.79, 1.26) | 0.85 (0.69, 1.05) | **0.55 (0.41, 0.73)** | 1.02 (0.81, 1.28) | 0.82 (0.66, 1.01) |
| Plant-based diet | 0.63 (0.33, 1.22) | 0.79 (0.44, 1.42) | 0.88 (0.54, 1.43) | **0.57 (0.40, 0.83)** | 0.98 (0.68, 1.40) | 0.90 (0.64, 1.27) | **0.66 (0.46, 0.94)** | 0.74 (0.52, 1.05) | 1.25 (0.91, 1.73) |
| Fast Foods | 0.81 (0.52, 1.26) | 1.00 (0.69, 1.45) | 0.89 (0.66, 1.21) | 0.91 (0.71, 1.15) | 1.08 (0.85, 1.36) | 0.84 (0.67, 1.04) | 1.12 (0.87, 1.43) | 1.16 (0.91, 1.49) | 0.94 (0.75, 1.18) |
| Red & Processed Meats | 0.77 (0.50, 1.19) | 0.84 (0.57, 1.23) | 0.79 (0.58, 1.09) | 0.98 (0.78, 1.24) | 1.03 (0.81, 1.31) | **0.78 (0.62, 0.97)** | 1.02 (0.80, 1.30) | 0.84 (0.65, 1.07) | **0.64 (0.51, 0.81)** |
| Sugar-sweetened beverages | 0.95 (0.56, 1.60) | 1.01 (0.65, 1.59) | 0.71 (0.48, 1.05) | 1.00 (0.75, 1.32) | 0.91 (0.69, 1.21) | 0.97 (0.73, 1.28) | 1.01 (0.75, 1.37) | 1.09 (0.81, 1.48) | 0.91 (0.69, 1.20) |
| Alcohol | 1.92 (0.87, 4.24) | 0.66 (0.33, 1.32) | 1.35 (0.73, 2.51) | 1.21 (0.80, 1.84) | 0.69 (0.44, 1.07) | 1.05 (0.68, 1.63) | **1.64 (1.15, 2.35)** | 1.31 (0.92, 1.86) | 1.20 (0.86, 1.68) |

AICR, American Institute for Cancer Research; DPP, Diabetes Prevention Program; WCRF, World Cancer Research Fund. Results with significant p-values (p<0.05) are bolded. Data shown are HR for incident diabetes per one-point positive change in Score.

^1^ The N included examining Score change between years 0 and 1 is slightly lower than the time-dependent Score analysis because some participants were missing Year 1 nutrition data but have subsequent years’ nutrition data. Score changes represent year 1 Scores-baseline Scores; thus, a larger value signifies an improvement in the Score.

^2^ Diabetes is defined based on the 1997 American Diabetes Association criteria: fasting plasma glucose ≥ 7 mmol/L (≥ 126 mg/dL) measured every six months or 2-hour plasma glucose ≥ 11.1 mmol/L (≥200 mg/dL) after a 75 g oral glucose load [cite: Diabetes Prevention Program Research Group 2009].

^3^ Year 0 through the end of the Diabetes Prevention Program represents a mean follow-up time of 3.2 years

^4^ Multivariate models adjusted for baseline covariates: age, sex, race/ethnicity, marital status, family history of diabetes, smoking status (never/former/current), years of education, total caloric intake, hormone therapy (females only), baseline risk score and the total score (change from baseline to Yr1) including the other 6 components (minus the component of interest)

^5^ DPPOS follow-up through January 2014 was included in the study, representing a mean follow-up time of 15 years

^6^ Multivariate models adjusted for baseline covariates: age, sex, race/ethnicity, marital status, family history of diabetes, smoking status (never/former/current), years of education, total caloric intake, hormone therapy (females only), baseline risk score and the time-dependent total score including the other 6 components (minus the component of interest)

## **Supplemental Table 5.** Hazard ratios and 95% confidence intervals for change in the components of the 2018 WCRF/AICR Score (0-7 points) and diabetes risk by treatment group^1,2^

|  | **Score change between years 0 and 1 on risk through end of DPP**^3,4^ | | | | **Score change between year 0 and 1 on risk through year 15**^4^**^,^**^5^ | | | **Time-dependent Score (years 0 to 15) on risk through year 15**^5^ | | |
| --- | --- | --- | --- | --- | --- | --- | --- | --- | --- | --- |
| **Component** | | **Lifestyle** | **Metformin** | **Placebo** | **Lifestyle** | **Metformin** | **Placebo** | **Lifestyle** | **Metformin** | **Placebo** |
| *Cases/N* | | *138/961* | *196/968* | *277/971* | *463/961* | *481/968* | *546/971* | *486/1044* | *515/1044* | *579/1059* |
| Body Weight & Physical Activity | | **0.35 (0.23, 0.53)** | 1.11 (0.80, 1.55) | 0.84 (0.64, 1.11) | **0.56 (0.45, 0.69)** | 0.88 (0.71, 1.10) | 0.87 (0.72, 1.07) | **0.48 (0.39, 0.58)** | 0.85 (0.70, 1.02) | **0.75 (0.63, 0.89)** |
| Nutrition Components | | 0.88 (0.69, 1.11) | 0.90 (0.72, 1.11) | 0.85 (0.71, 1.01) | 0.92 (0.81, 1.05) | 0.98 (0.86, 1.12) | **0.86 (0.76, 0.98)** | 1.02 (0.90, 1.16) | 1.00 (0.88, 1.14) | 0.89 (0.80, 1.01) |
| Score without Body Weight | | 0.85 (0.68, 1.06) | 0.98 (0.81, 1.20) | **0.85 (0.73, 0.99)** | 0.92 (0.81, 1.04) | 0.99 (0.88, 1.11) | **0.86 (0.77, 0.95)** | 0.95 (0.85, 1.07) | 1.00 (0.89, 1.12) | **0.88 (0.79, 0.98)** |

AICR, American Institute for Cancer Research; DPP, Diabetes Prevention Program; WCRF, World Cancer Research Fund. Results with significant p-values (p<0.05) are bolded. Data shown are HR for incident diabetes per one-point positive change in Score.

^1^ The N included examining Score change between years 0 and 1 is slightly lower than the time-dependent Score analysis because some participants were missing Year 1 nutrition data but have subsequent years’ nutrition data. Score changes represent Year 1 Scores-baseline Scores; thus, a larger value signifies an improvement in the Score.

^2^ Diabetes is defined based on the 1997 American Diabetes Association criteria: fasting plasma glucose ≥ 7 mmol/L (≥ 126 mg/dL) measured every six months or 2-hour plasma glucose ≥ 11.1 mmol/L (≥200 mg/dL) after a 75 g oral glucose load [cite: Diabetes Prevention Program Research Group 2009].

^3^ Year 0 through the end of the Diabetes Prevention Program represents a mean follow -up time of 3.2 years

^4^ Models adjusted for baseline covariates: age, sex, race/ethnicity, marital status, family history of diabetes, smoking status (never/former/current), years of education, total caloric intake, hormone therapy (females only), baseline risk score and the total score (change from baseline to Yr1) including the other 6 components (minus the component of interest)

^5^ DPPOS follow-up through January 2014 was included in the study, representing a mean follow-up time of 15 years
